# Treatment regimens for neuromyelitis optica spectrum disorder attacks: a retrospective cohort study

**DOI:** 10.1186/s12974-022-02420-2

**Published:** 2022-03-02

**Authors:** Stanislas Demuth, Maxime Guillaume, Bertrand Bourre, Jonathan Ciron, Hélène Zephir, Yoann Sirejacob, Anne Kerbrat, Christine Lebrun-Frenay, Caroline Papeix, Laure Michel, David Laplaud, Sandra Vukusic, Elisabeth Maillart, Mikael Cohen, Bertrand Audoin, Romain Marignier, Nicolas Collongues, for the NOMADMUS Study Group

**Affiliations:** 1 Department of Neurology, Strasbourg University Hospital, Strasbourg, France; 2 CHU de Rouen / Rouen University Hospital, 76000 Rouen, France; 3 Department of Neurology, CRC-SEP, CHU Toulouse, 31059 Toulouse Cedex 9, France; 4 Institut Toulousain des Maladies infectieuses et Inflammatoires (Infinity), INSERM UMR1291 - CNRS UMR5051, Université Toulouse III, Toulouse, France; 5 Department of Neurology, Inserm U 1172, University Hospital of Lille, University of Lille, Lille, France; 6 Department of Clinical Research, Rouen University Hospital, 76000 Rouen, France; 7 Department of Neurology, Rennes University Hospital, 35033 Rennes, France; 8 Department of Neurology, CHU de Nice, UR2CA-URRIS, Nice Côte d’Azur University, Nice, France; 9 Department of Neurology, AP-HP, Pitié-Salpêtrière Hospital, 75013 Paris, France; 10 Service de Neurologie, CHU Nantes, Nantes, France; 11 Department of Neurology, Hôpital Neurologique, Hospices Civils de Lyon, Bron, France; 12 Department of Neurology, University Hospital of Marseille, Marseille, France; 13 Aix-Marseille University, CRMBM UMR 7339, CNRS, Marseille, France; 14 Department of Neurology, Hôpital Wertheimer, HCL, Bron, France; 15 Department of Neurology, CHU Poitiers, 86021 Poitiers, France; 16 CRTI-InsermU1064, Nantes, France; 17 CHU de Nantes, Université de Nantes, Nantes, France; 18 Service de neurologie, sclérose en plaques, pathologies de la myéline et neuro-inflammation, and Centre de Référence des Maladies Inflammatoires Rares du Cerveau et de la Moelle, Hôpital Neurologique Pierre Wertheimer, Hospices Civils de Lyon, 69677 Lyon/Bron, France; 19 INSERM 1028 et CNRS UMR5292, Centre Des Neurosciences de Lyon, 69003 Lyon, France; 20 Université Claude Bernard Lyon 1, 69000 Lyon, France; 21 NeuroBioTec - Hôpital Neurologique Pierre Wertheimer, 59 boulevard Pinel, Bron, France

**Keywords:** Neuromyelitis optica, Therapeutics, MOGAD, Corticosteroids, Plasma exchanges, Relapses

## Abstract

**Background:**

Neuromyelitis optica spectrum disorder (NMOSD) attacks require an urgent probabilistic anti-inflammatory therapeutic strategy. As inadequately treated attacks result in disability, there is a need to identify the optimal attack-treatment regimen. Our study aimed to identify predictors of outcome after a first attack in patients with an NMOSD presentation and propose the best treatment strategy.

**Methods:**

We performed a retrospective cohort study on the French national NMOSD registry (NOMADMUS), a nested cohort of the French multiple sclerosis observatory (OFSEP) recruiting patients with NMOSD presentations in France. We studied the first attack for any independent locations of clinical core characteristic of NMOSD, in treatment-naïve patients. The primary outcome was the evolution of the Expanded Disability Status Scale (EDSS) score at 6 months, stratified in two ways to account for recovery (return to baseline EDSS score) and treatment response (classified as “good” if the EDSS score decreased by ≥ 1 point after a nadir EDSS score ≤ 3, or by ≥ 2 points after a nadir EDSS score > 3). We used ordinal logistic regression to infer statistical associations with the outcome.

**Results:**

We included 211 attacks among 183 patients (104 with anti-AQP4 antibodies, 60 with anti-MOG antibodies, and 19 double seronegative). Attack treatment regimens comprised corticosteroids (*n* = 196), plasma exchanges (PE; *n* = 72) and intravenous immunoglobulins (*n* = 6). Complete recovery was reached in 40 attacks (19%) at 6 months. The treatment response was “good” in 134 attacks (63.5%). There was no improvement in EDSS score in 50 attacks (23.7%). MOG-antibody seropositivity and short delays to PE were significantly and independently associated with better recovery and treatment response.

**Conclusions:**

We identified two prognostic factors: serostatus (with better outcomes among MOG-Ab-positive patients) and the delay to PE. We, therefore, argue for a more aggressive anti-inflammatory management of the first attacks suggesting an NMOSD presentation, with the early combination of PE with corticosteroids.

## Background

Neuromyelitis optica spectrum disorder (NMOSD) attacks are more damaging than multiple sclerosis (MS) attacks and require more aggressive treatments [1–3]. According to the most recent diagnostic criteria [4], NMOSD may be associated with anti-aquaporin 4 (AQP4) antibodies targeting astrocytes, with anti-myelin oligodendrocyte glycoprotein (MOG) antibodies targeting oligodendrocytes or with double seronegative status (NMOSD–DN, i.e., seronegative for AQP4 and MOG antibodies). However, AQP4-Ab-positive patients and MOG-Ab-positive patients have different immunopathologic and clinical courses and the nosography is currently evolving rapidly. Indeed, patients positive for MOG-Ab included in the NMOSD set of criteria have recently been individualized as having MOG-associated diseases (MOGAD) [5, 6], and there is some debate about the classification of MOGAD in NMOSD [7]. Despite this, AQP4-Ab-positive and MOG-Ab-positive patients can have a similar initial presentation, corresponding to an NMOSD phenotype; Such phenotypes have been well described [4]: longitudinally extensive transverse myelitis, severe optic neuritis (ON), area postrema/diencephalic/brainstem or encephalic syndrome. The first attack of these patients must, therefore, be considered diagnostically uncertain until the results of the serological status are known. During this period, if an NMOSD phenotype is observed, there is an urgent need to instigate a powerful anti-inflammatory therapeutic strategy. Consequently, despite MOGAD having a broader and less clearly defined spectrum of manifestations than classical NMOSD as defined by the IPND criteria [4, 8–10], we focused on NMOSD phenotypical first presentations, and thus used the term NMOSD–MOG+ in this report.

Attack treatment strategy in NMOSD relies on various combinations of high-dose intravenous corticosteroids and apheresis methods including plasma exchanges (PE), immunoadsorption (IA) or intravenous immunoglobulins (IVIG). As inadequately treated attacks result in disability [11, 12], there is a need to identify the optimal attack treatment regimen. There is no randomized clinical trial for the treatment of NMOSD relapses but there have been efforts to reach a consensus [13–15]. Previous retrospective studies suggest the superiority of PE in AQP4-Ab-positive patients (NMOSD–AQP4+), with better outcomes when initiated early [11, 16–21]. However, most of these studies did not address the specificities of NMOSD–DN or NMOSD–MOG+ and suffered from potential confusion with the effects of immunosuppressive treatments at the time of the attacks. Therefore, we investigated in naïve-treatment patients the first attacks for any given location related to an NMOSD presentation and looked for predictive factors to identify the best treatment strategy.

## Methods

### Ethics, consent and permissions

We performed a retrospective cohort study on the French national NMOSD database NOMADMUS (ClinicalTrials.gov Identifier: NCT02850705), a nested cohort of the French MS observatory (Observatoire Français de la Sclérose En Plaques; OFSEP) recruiting patients with NMOSD presentations in France. Although the NOMADMUS registry is not mandatory, our cohort can be considered exhaustive as the detection of anti-AQP4 and anti-MOG antibodies is centralized in Lyon for suspected cases in France. Moreover, all suspected cases are reviewed by a multidisciplinary board of NMOSD experts. All patients gave their written informed consent and data collection was approved by the national ethical authority (Commission Nationale de l'Informatique et des Libertés; CNIL; registration number 914066v2). Data are regularly completed and cleaned by clinical research assistants on site and affiliated to OFSEP.

### Inclusion criteria and data collection

We first screened the NOMADMUS database for patients meeting either the 2015 diagnostic criteria [4] or presenting with a first attack suggestive of NMOSD (transverse myelitis, severe ON or an area postrema/diencephalic/brainstem or encephalic syndrome). We defined an attack as a new neurological deficit with EDSS score worsening lasting at least 24 h, in the absence of hyperthermia, and separated from any previous episode by at least 30 days. We included all such NMOSD presentations in treatment-naïve adult patients (i.e., aged over 18 years, with no immunosuppressive treatment during the previous 6 months). If the patient was still treatment naïve, further attacks were included. To avoid confusion with a residual disability, we only investigated the first attack for any given location among myelitis, ON, or acute brainstem or diencephalic syndrome, and symptomatic cerebral syndrome (encephalitis). Clinical follow-up had to be a minimum of 6 months. The patients were tested for anti-AQP4 and anti-MOG autoantibodies using a cell-based assay, which allowed us to define three groups of patients: NMOSD–AQP4+, NMOSD–MOG+, and NMOSD–DN. All NMOSD–DN patients were tested for both antibodies and fulfilled the 2015 diagnostic criteria at the time of screening. The extraction from the NOMADMUS database yielded the list of patients per investigation site. To ensure a high standard of quality of our results according to disability and time to treatment, all clinical files of this list were checked locally by a “flying” investigator. Data were then collected on-site from clinical records: demographic data, the date and characteristics of the different attacks, clinical evolution, and the timing and posology of attack treatments. In addition, all MRI and fundoscopic examinations were collected if they were performed less than 3 months after the attack onset. The attack treatment regimens were structured as a succession of therapeutic lines of either high dose corticosteroids, or PE, or IVIG. The inflammatory lesions were characterized by MRI according to the evocative findings of the 2015 IPND criteria [4].

### Outcome

**Fig. 1:**
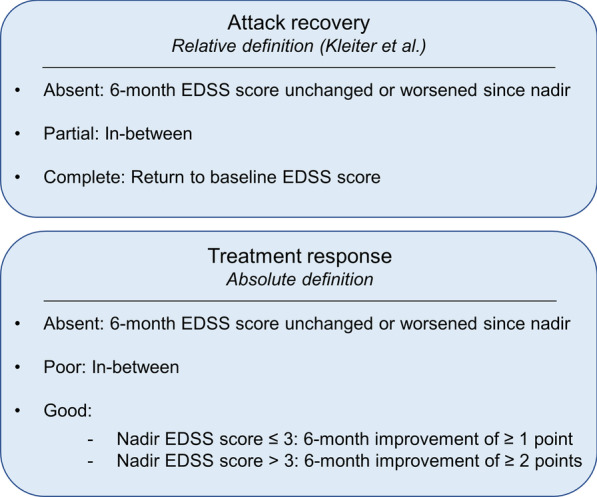
We classified the recovery as “complete”, “partial”, or “absent” using a relative and an absolute definition. The relative definition was that proposed by Kleiter et al. [17], classifying the recovery. The absolute definition described the treatment response, based on the EDSS improvement at 6 months

The outcome of interest was the recovery from the attack, assessed by the evolution of the Expanded Disability Status Scale (EDSS) score between the nadir of the attack and 6 months later [22]. If the EDSS score was not available in the clinical record, we scored the EDSS retrospectively according to the neurostatus assessment [22] when sufficient clinical data were available at nadir, at 6 months, and at last follow-up. The baseline EDSS score was the last known EDSS score before the attack and was considered to be 0 in the case of a first attack. Patients with insufficient clinical data to score the EDSS at these timepoints were discarded. We classified the recovery as “complete,” “partial,” or “absent” using a relative and an absolute definition (Fig. 1). The relative definition was that proposed by Kleiter et al. [17], classifying the recovery as “complete” if the 6-month EDSS score reached the score before the attack, “partial” if recovery was incomplete, and “absent” if there was no improvement or a clinical deterioration. The absolute definition described the treatment response, based on the EDSS score improvement at 6 months, classifying the response as “good” if the 6-month EDSS score showed a decrease of ≥ 1 point in the case of a nadir EDSS score ≤ 3 or a reduction of ≥ 2 points in the case of a nadir EDSS score > 3. Treatment response was classified as “poor” if the EDSS score decrease was slighter and as “absent” if the EDSS score remained unchanged or if it worsened.

### Statistical analysis

Statistical analyses were performed using the software R (version 4.0.3). Categorical variables are summarized as count and percentages, and quantitative variables as mean and standard deviation (SD), except for EDSS scores, which are presented as median and interquartile range (IQR). We compared categorical variables with *χ*^2^ tests and quantitative variables with Kruskal–Wallis tests (or ANOVA tests when the normality and variance homoscedasticity assumptions were met according to Shapiro’s and Bartlett’s tests, respectively). We used the “polr” function of the R package “MASS” to build ordinal logistic regression models [23] to infer the effect of the dependent variables on the outcome: one model with the relative definition and one model with the absolute definition as outcomes. Age, gender, and variables associated with a *p* value < 0.20 in the univariate analysis were included in the multivariate analysis. Underrepresented variables hindering model convergence were excluded (*n* < 10) as well as collinear variables (for instance: delay to the second therapeutic line versus delay to plasma exchanges). Subcategorical variables presenting the characteristics of attack locations could not be included in the model and were substituted by higher level variables, such as the attack location. We considered *p*-values < 0.05 as statistically significant.

### Data availability statement

Anonymized data and R code will be shared by request from any qualified investigator.

## Results

Among the French national NMOSD cohort NOMADMUS, 374 patients met the inclusion criteria. Our on-site data collection found a complete data set including posology and time to attack treatment, EDSS score at nadir and at 6 months or found enough data to score them retrospectively in 183 patients from 11 centers (Table 1). This represents 211 attacks; 179 attacks were the first one in the patient’s history (84.8%). The others affected an independent location. Almost all attacks were confirmed on MRI (*n* = 202; 95.7%). A majority of patients were female (*n* = 121; 68.9%). The mean age at first attack was 38.5 years (range: 13–80). Among the 183 patients, 104 had anti-AQP4 antibodies (56.8%), 60 had anti-MOG antibodies (32.8%), and 19 were NMOSD double seronegative (10.4%). Attacks were mostly isolated myelitis (*n* = 93; 44%) and isolated ON (*n* = 74; 35%), with 16 combined attacks of neuromyelitis optica (NMO; 8%) (Table 2). The median nadir EDSS score was 5.00 (range: 1–9.5). Among the myelitis attacks, 98 were longitudinally extensive and transverse at MRI (76%). Among the ON attacks, 43 were associated with papilledema (44.8%), 39 were bilateral (40.6%), and 33 were extensive at MRI (34.4%).

**Table 1:** Characteristics of NMOSD attacks

	NMOSD–AQP4+ (*N* = 121)	NMOSD–MOG+ (*N* = 67)	NMOSD–DN (*N* = 23)	*P*-value	Total (*N* = 211)
Sex (female)	103 (85%)	30 (45%)	14 (61%)	< 0.001	147 (70%)
Age at attack	40.8 (15.5)	35.6 (14.5)	33.8 (12.8)	0.021	38.4 (15.1)
Optic neuritis	42 (35%)	42 (63%)	12 (52%)	< 0.001	96 (45%)
Papilloedema	13 (11%)	28 (42%)	2 (9%)	0.00213	43 (45%)
Bilateral	13 (11%)	20 (30%)	6 (26%)	0.238	39 (41%)
Extensive	14 (12%)	14 (21%)	5 (22%)	0.562	33 (34%)
Myelitis	82 (68%)	27 (40%)	18 (78%)	< 0.001	127 (60%)
LETM	64 (53%)	19 (28%)	15 (65%)	0.365	98 (76%)
Brainstem	14 (12%)	7 (10%)	4 (17%)	0.667	25 (12%)
Encephalic syndrome	3 (2%)	3 (4%)	0 (0%)	0.502	6 (3%)
ADEM	3 (2%)	0 (0%)	0 (0%)	0.322	3 (1%)
Location profile					
Isolated myelitis	66 (55%)	17 (25%)	10 (43%)	< 0.001	93 (44%)
Isolated optic neuritis	34 (28%)	35 (52%)	4 (17%)		73 (35%)
Neuromyelitis optica	6 (5%)	5 (7%)	5 (22%)		16 (8%)
Other	15 (12%)	10 (15%)	4 (17%)		29 (14%)
Nadir EDSS score (median; IQR)	5.00 (4.50)	4.00 (2.50)	6.00 (3.00)	0.106	5.00 (4.00)
EDSS score at 6 months (median; IQR)	3.50 (3.00)	1.50 (2.75)	3.50 (4.00)	< 0.001	2.50 (4.00)
EDSS score change (median; IQR)	− 1.50 (3.00)	− 3.00 (2.50)	− 1.00 (3.00)	0.00128	− 2.00 (2.50)
Number of therapeutic lines	1.53 (0.673)	1.24 (0.605)	1.65 (0.832)	0.0134	1.45 (0.685)
Treatments					
Corticosteroids	116 (96%)	60 (90%)	20 (87%)	0.137	196 (93%)
PE	44 (36%)	19 (28%)	9 (39%)	0.468	72 (34%)
IVIG	4 (3%)	1 (1%)	1 (4%)	0.696	6 (3%)
Delay (days) to first line	15.3 (18.3)	11.9 (13.1)	16.4 (26.1)	0.828	14.3 (17.9)
Delay (days) to second line	27.7 (18.1)	20.3 (13.3)	41.1 (40.8)	0.213	27.8 (22.1)
Delay (days) to third line	36.3 (20.4)	NA	88.0 (54.7)	0.074	53.5 (41.6)
Delay (days) to PE	26.5 (22.4)	19.8 (14.2)	38.3 (52.3)	0.687	26.3 (26.4)

Quantitative variables are presented as mean (SD), except EDSS scores, which are presented as median (IQR). Categorical variables are presented as count (percentage)

NMOSD–AQP4+: NMOSD attacks with anti-AQP4 antibodies; NMOSD–MOG+: NMOSD attacks with anti-MOG antibodies; NMOSD–DN: double seronegative NMOSD attacks; LETM: longitudinally extensive transverse myelitis; ADEM: acute disseminated encephalomyelitis; EDSS: Expanded Disability Status Scale; PE: plasma exchanges; IVIG: intravenous immunoglobulins; NA: not applicable

*P*-values reflect Kruskal–Wallis statistical test results

**Table 2:** Location profile of NMOSD attacks

Location profile	NMOSD–AQP4+ (*N* = 121)	NMOSD–MOG+ (*N* = 67)	NMOSD–DN (*N* = 23)	Total (*N* = 211)
Isolated myelitis	66 (71.0%)	17 (18.3%)	10 (10.8%)	93 (44%)
Isolated optic neuritis	34 (46.6%)	35 (47.9%)	4 (5.5%)	73 (35%)
Isolated brainstem	5 (71.4%)	2 (28.6%)	0 (0%)	7 (3.3%)
Isolated encephalitis	0 (0%)	2 (100%)	0 (0%)	2 (0.9%)
Myelitis + optic neuritis (Neuromyelitis optica)	6 (37.5%)	5 (31.3%)	5 (31.3%)	16 (8%)
Optic neuritis + brainstem	0 (0%)	0 (0%)	1 (100%)	1 (0.5%)
Optic neuritis + encephalitis	0 (0%)	1 (100%)	0 (0%)	1 (0.5%)
Myelitis + encephalitis	1* (100%)	0 (0%)	0 (0%)	1 (0.5%)
Myelitis + brainstem	6 (54.5%)	4 (36.4%)	1 (9.1%)	11 (5.2%)
Myelitis + optic neuritis + brainstem	1 (25.0%)	1 (25.0%)	2 (50.0%)	4 (1.9%)
Myelitis + brainstem + encephalitis	1* (100%)	0 (0%)	0 (0%)	1 (0.5%)
Myelitis + optic neuritis + brainstem + encephalitis	1* (100%)	0 (0%)	0 (0%)	1 (0.5%)

Neuromyelitis optica spectrum disorder presentations and their serotypes. Nine attacks were multifocal, including 3 cases of NMOSD–AQP4+ acute demyelinating encephalomyelitis (*). Categorical variables are presented as count (percentage). Percentages are in the column axis for the “Total” column and in the row axis for the others

NMOSD–AQP4+: NMOSD attacks with anti-AQP4 antibodies; NMOSD–MOG+: NMOSD attacks with anti-MOG antibodies; NMOSD–DN: double seronegative NMOSD attacks

The majority of patients in the NMOSD–AQP4+ and NMOSD–DN groups were female (85% and 61%, respectively) but the sex ratio was more balanced in the NMOSD–MOG+ group (45% female). The mean age at first attack was higher in the NMOSD–AQP4+ group (40.8 years, versus 35.6 years in the NMOSD–MOG+ group and 33.8 years in the NMOSD–DN group; *p* = 0.021). Myelitis was the most frequent location in the NMOSD–AQP4+ and NMOSD–DN groups (Table 2; 68% and 78%, respectively). ON was the most frequent presentation in the NMOSD–MOG+ group. Three cases of acute disseminated encephalomyelitis (ADEM) were reported (1.4%), all in the NMOSD–AQP4+ group. The nadir EDSS score was similar for all serogroups (Table 1; *p* = 0.106).

**Fig. 2:**
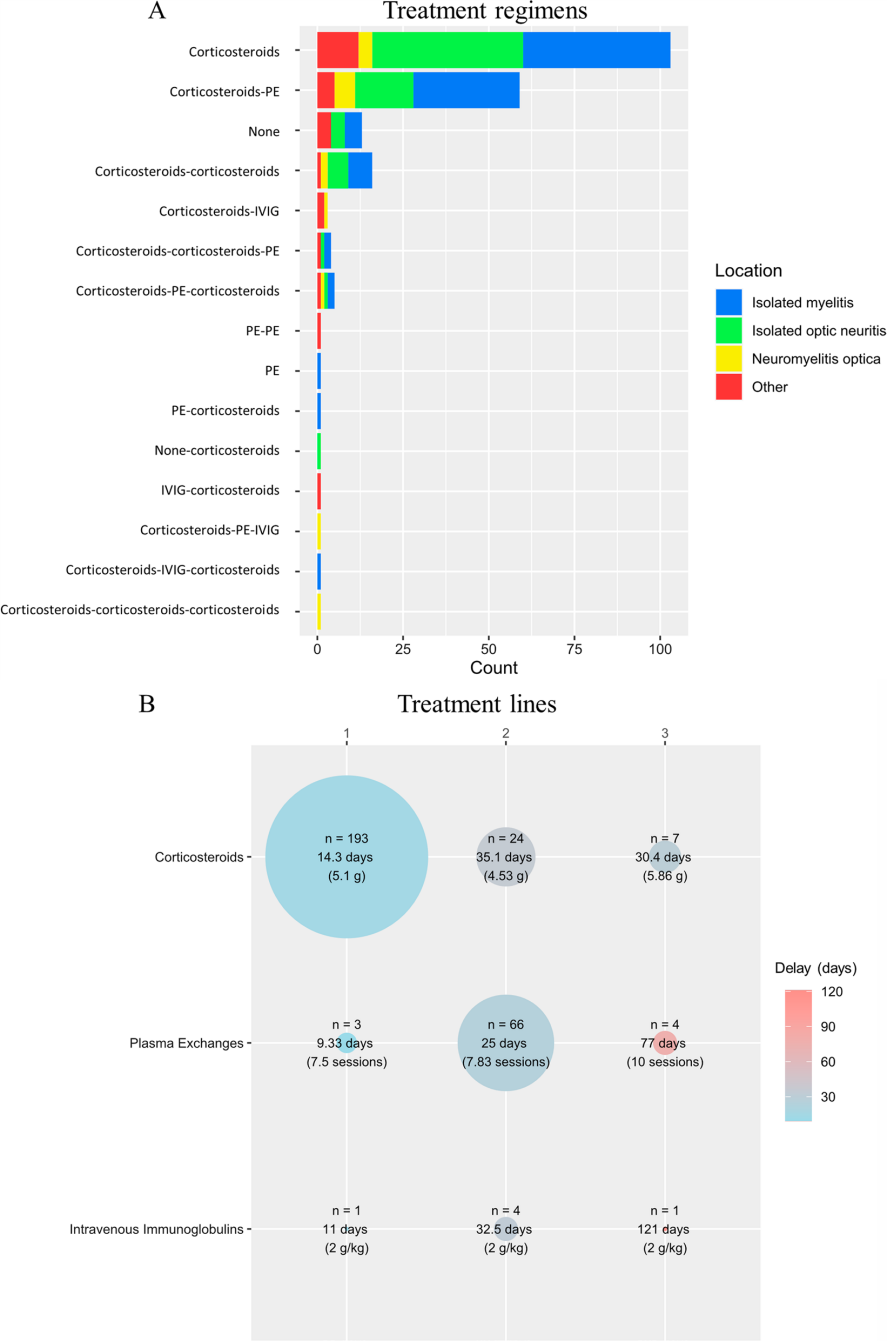
Treatment regimens structured as three therapeutic lines (**A**) and corticosteroids, PE, and IVIG usage at each line (**B**). The size of each circle is proportional to the number of attacks. The color corresponds to the mean delay. *PE* plasma exchange, *IVIG* intravenous immunoglobulins

Patients were treated with various regimens of corticosteroids, PE, or IVIG (Fig. 2). The mean delay between the onset of symptoms and the start of treatment was 14.3 days (SD = 17.9 days). Corticosteroids were the most frequent first-line treatment (*n* = 193; 91.9%), with a mean delay of 14.3 days, and PE was the most frequent second-line treatment (*n* = 66; 70.2%), with a mean delay of 25.0 days (overall mean = 26.3 days, overall median = 16 days, whatever the treatment line). Fourteen attacks were untreated (6.6%). Ninety-four patients had a combined regimen (44.5%), corticosteroids followed by PE being the most frequent combination (Fig. 2). A second line of treatment was required for 44.5% of attacks and a third line for 5.7%. The treatment regimen was similar whatever the location of the attack (Fig. 2) and the serogroup (Table 1), except for a lower mean number of treatment lines for the NMOSD–MOG + group (mean = 1.24, versus 1.65 for the NMOSD–DN group and 1.53 for the NMOSD–AQP4+ group; *p* = 0.013).

**Fig. 3:**
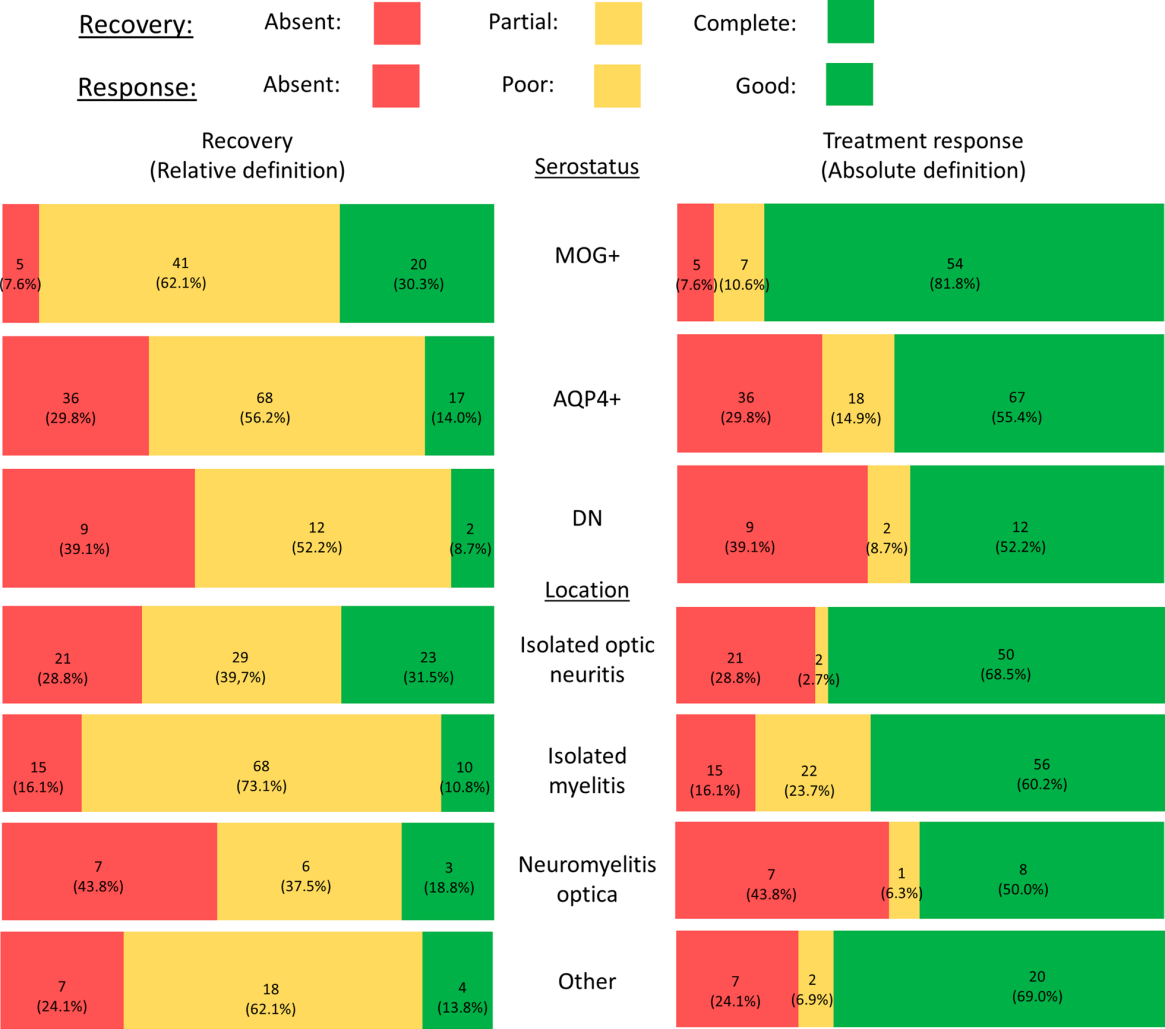
6-Month clinical outcome, presented as bar plots. We classified the recovery as “complete”, “partial”, or “absent” using a relative and an absolute definition. The relative definition was that proposed by Kleiter et al. [17], classifying the recovery as “complete” if the 6-month EDSS score reached the score before the attack, “partial” if recovery was incomplete, and “absent” if there was no improvement or a clinical deterioration. The absolute definition described the treatment response, based on the EDSS improvement at 6 months, classifying the response as “good” if the 6-month EDSS score showed a decrease of ≥ 1 point in the case of nadir EDSS score ≤ 3 or a reduction of ≥ 2 points in the case of a nadir EDSS score > 3. Treatment response was classified as “poor” if the EDSS score decrease was slighter and as “absent” if the EDSS score remained unchanged or if it worsened. Counts and percentages are shown for each bar. AQP4+: NMOSD–AQP4+; MOG+: NMOSD–MOG+; DN: NMOSD–DN (double seronegative)

At 6 months, the median EDSS score was 2.50 (range: 0–10), with a median improvement of 2.00 points (range: 3.0-point deterioration–8.0-point improvement). NMOSD–MOG+ patients showed the greatest improvement (Table 1), with a median 6-month EDSS score of 1.50 (*p* < 0.001) and a median improvement of 3.00 points (*p* = 0.001). Recovery was complete after 40 attacks (19.0%), according to the relative definition (Tables 3 and 4, Fig. 3), and the response was good after 134 attacks (63.5%), according to the absolute definition (Tables 3 and 5, Fig. 3). According to both definitions, there was no improvement (absence of recovery or response) after 50 attacks (23.7%). Patients in the NMOSD–MOG+ group reached complete recovery or a good response more frequently than patients in the NMOSD–AQP4+ and NMOSD–DN groups (respectively: 31.3% versus 14.0% and 8.7% for recovery; 82.1% versus 55.4% and 52.2% for treatment response; Fig. 3). We found these differential associations when corticosteroids or PE were included in the therapeutic regimen (Table 3). At 1-year follow-up, we recorded the introduction of a disease modifying treatment after 138 attacks of all 211 attacks (65.4%): by serogroup, after 91 of 121 attacks (75.2%) in the NMOSD–AQP4+ group, after 32 of 67 attacks (47.8%) in the NMOSD–MOG+ group and after 15 of 23 attacks (65.2%) in the NMOSD–DN group. Among the 73 attacks without the subsequent introduction of a disease modifying treatment, 65 (89.0%) were the patient’s first attack: by serogroup these comprised 26 of 30 attacks in the NMOSD–AQP4+ group, 32 of 35 attacks in the NMOSD–MOG+ group and 7 of 8 attacks in the NMOSD–DN group.

**Table 3:** Proportion of complete recovery and responses per serotype and treatment

	NMOSD–AQP4+ (*N* = 121)	NMOSD–MOG+ (*N* = 67)	NMOSD–DN (*N* = 23)	Total (*N* = 211)
Relative definition				
Included in the therapeutic regimen				
Corticosteroids (*N* = 196)	14 (12.1%)	20 (33.3%)	2 (10.0%)	36 (18.4%)
PE (*N* = 72)	2 (4.5%)	4 (21.1%)	1 (11.1%)	7 (9.7%)
IVIG (*N* = 6)	0 (0%)	0 (0%)	0 (0%)	0 (0%)
Absolute definition				
Included in the therapeutic regimen				
Corticosteroids (*N* = 196)	63 (54.3%)	51 (85.0%)	10 (50.0%)	124 (63.3%)
PE (*N* = 72)	23 (52.3%)	17 (89.5%)	4 (44.4%)	44 (61.1%)
IVIG (*N* = 6)	0 (0%)	0 (0%)	0 (0%)	0 (0%)

Complete recoveries according to the relative definition (upper table) and the absolute definition (lower table) are presented as counts (proportion). The relative definition was that proposed by Kleiter et al. [17], classifying the recovery as “complete” if the 6-month EDSS score reached the score before the attack, “partial” if recovery was incomplete, and “absent” if there was no improvement or a clinical deterioration. The absolute definition described the treatment response, based on the EDSS improvement at 6 months, classifying the response as “good” if the 6-month EDSS score showed a decrease of ≥ 1 point in the case of nadir EDSS score ≤ 3 or a reduction of ≥ 2 points in the case of a nadir EDSS score > 3. Treatment response was classified as “poor” if the EDSS score decrease was slighter and as “absent” if the EDSS score remained unchanged or if it worsened

NMOSD–AQP4+: NMOSD attacks with anti-AQP4 antibodies; NMOSD–MOG+: NMOSD attacks with anti-MOG antibodies; NMOSD–DN: double seronegative NMOSD attacks; PE: plasma exchanges; IVIG: intravenous immunoglobulins

**Table 4:** Predictors of recovery (relative definition)

	Recovery (relative definition)					
	Complete (*N* = 40)	Partial (*N* = 121)	Absent (*N* = 50)	Total (*N* = 211)	Multivariate analysis	
					ORa	*P*-value
Sex (female)*	25 (62%)	83 (69%)	39 (78%)	147 (70%)	0.42 [0.096; 1.78]	0.24
Age at attack*	33.7 (12.2)	39.9 (15.9)	38.7 (14.9)	38.4 (15.1)	1.01 [0.97; 1.06]	0.53
Serostatus***						
AQP4+	17 (42%)	68 (56%)	36 (72%)	121 (57%)		
MOG+***	21 (52%)	41 (34%)	5 (10%)	67 (32%)	0.058 [0.078; 0.35]	0.0042
DN	2 (5%)	12 (10%)	9 (18%)	23 (11%)	0.88 [0.13; 6.49]	0.91
Location profile						
Isolated myelitis	10 (25%)	68 (56%)	15 (30%)	93 (44%)		
Isolated optic neuritis	23 (58%)	29 (24%)	21 (42%)	73 (35%)	3.26 [0.44; 27.7]	0.26
Neuromyelitis optica	3 (8%)	6 (5%)	7 (14%)	16 (8%)	8.82 [1.25; 80.2]	0.041
Other	4 (10%)	18 (15%)	7 (14%)	29 (14%)	0.76 [0.092; 5.58]	0.79
Nadir EDSS score*	3.20 (1.90)	5.88 (2.09)	4.46 (1.93)	5.04 (2.27)	0.96 [0.62; 1.50]	0.87
Number of therapeutic lines**	1.13 (0.563)	1.53 (0.686)	1.54 (0.706)	1.45 (0.685)	0.45 [0.082; 2.28]	0.35
Treatments						
Corticosteroids	36 (90%)	113 (93%)	47 (94%)	196 (93%)		
PE*	7 (18%)	47 (39%)	18 (36%)	72 (34%)		
IVIG**	0 (0%)	2 (2%)	4 (8%)	6 (3%)		
Delay (days) to first line	13.6 (17.8)	13.3 (14.6)	17.3 (24.3)	14.3 (17.9)		
Delay (days) to second line**	16.3 (8.56)	25.0 (19.4)	39.0 (27.6)	27.8 (22.1)		
Delay (days) to third line*	NA	39.1 (20.8)	82.3 (60.6)	53.5 (41.6)		
Delay (days) to PE**	16.3 (9.25)	19.8 (14.3)	47.1 (41.1)	26.3 (26.4)	1.04 [1.01; 1.07]	0.012

Quantitative variables are presented as mean (SD). Categorical variables are presented as count (percentage)

*DN* double seronegative NMOSD attacks, *EDSS* Expanded Disability Status Scale, *PE* plasma exchanges, *OR* odds ratio, *ORa* adjusted odds ratio, *NA* not applicable

Univariate analysis: *p-value < 0.2; **p-value < 0.05; ***p-value < 0.001

**Table 5:** Predictors of treatment response (absolute definition)

	Treatment response (absolute definition)					
	Good (*N* = 134)	Poor (*N* = 27)	Absent (*N* = 50)	Total (*N* = 211)	Multivariate analysis	
					ORa	*P*-value
Sex (female)*	89 (66%)	19 (70%)	39 (78%)	147 (70%)	0.24 [0.047; 1.05]	0.07
Age at attack*	36.7 (14.7)	46.3 (15.8)	38.7 (14.9)	38.4 (15.1)	1.01 [0.97; 1.06]	0.49
Serostatus***						
AQP4+	67 (50%)	18 (67%)	36 (72%)	121 (57%)		
MOG+***	55 (41%)	7 (26%)	5 (10%)	67 (32%)	0.048 [0.0041; 0.35]	0.008
DN	12 (9%)	2 (7%)	9 (18%)	23 (11%)	1.25 [0.20; 7.75]	0.81
Location profile*						
Isolated myelitis	56 (42%)	22 (81%)	15 (30%)	93 (44%)		
Isolated optic neuritis	50 (37%)	2 (7%)	21 (42%)	73 (35%)	2.56 [0.55; 12.1]	0.23
Neuromyelitis optica*	8 (6%)	1 (4%)	7 (14%)	16 (8%)	4.83 [0.83; 32.6]	0.089
Other	20 (15%)	2 (7%)	7 (14%)	29 (14%)	0.23 [0.0099; 1.99]	0.24
Nadir EDSS	5.04 (2.38)	6.11 (1.96)	4.46 (1.93)	5.04 (2.27)		
Number of therapeutic lines	1.44 (0.678)	1.37 (0.688)	1.54 (0.706)	1.45 (0.685)		
Treatments						
Corticosteroids	124 (93%)	25 (93%)	47 (94%)	196 (93%)		
PE	44 (33%)	10 (37%)	18 (36%)	72 (34%)		
IVIG*	2 (1%)	0 (0%)	4 (8%)	6 (3%)		
Delay (days) to first line*	12.8 (14.3)	16.4 (20.3)	17.3 (24.3)	14.3 (17.9)		
Delay (days) to second line**	24.9 (19.8)	19.0 (9.50)	39.0 (27.6)	27.8 (22.1)		
Delay (days) to third line	42.7 (19.6)	NA	82.3 (60.6)	53.5 (41.6)		
Delay (days) to PE**	19.8 (14.8)	17.2 (7.79)	47.1 (41.1)	26.3 (26.4)	1.03 [1.01; 1.07]	0.024

Quantitative variables are presented as mean (SD). Categorical variables are presented as count (percentage)

*DN* double seronegative NMOSD attacks, *EDSS* Expanded Disability Status Scale, *PE* plasma exchanges, *OR* odds ratio, *ORa* adjusted odds ratio, *NA* not applicable

Univariate analysis: *p value < 0.2; **p value < 0.05; ***p value < 0.001

Using the relative classification of improvement as recovery (Table 4), our inferential statistical analysis showed that better outcomes were significantly associated with NMOSD–MOG+ patients (OR = 0.29 versus NMOSD–AQP4+ patients; 95% CI [0.15; 0.53]; *p* = 0.0042). Poorer recoveries were significantly associated with the requirement for more treatment lines (OR = 1.69; 95% CI [1.15; 2.51]; *p* = 0.009), delayed PE (OR = 1.04 per day; 95% CI [1.02; 1.07]; *p* = 0.0023) and extensive ON (OR = 3.51; 95% CI [1.46; 8.8]; *p* = 0.007). Recovery did not significantly correlate with bilateral ON (OR = 1.68; 95% CI [0.78; 3.66]; *p* = 0.19), nor ON associated with papillary edema (OR = 0.58; 95% CI [0.26; 1.28]; *p* = 0.18), nor longitudinally extensive transverse forms of myelitis (OR = 1.78; 95% CI [0.69; 4.68]; *p* = 0.24). At the multivariate analysis, MOG-Ab-positivity was significantly and independently associated with better recoveries (OR = 0.058 versus AQP4-Ab+ patients; 95% CI [0.0078; 0.35]; *p* = 0.0042) and delayed PE with poorer recoveries (OR = 1.04 per day; 95% CI [1.01; 1.07]; *p* = 0.012). Combined ON and transverse myelitis attacks (NMO) were independently associated with poorer recovery in comparison to the other presentations (OR = 8.82 versus isolated transverse myelitis; 95% CI [1.25; 80.2]; *p* = 0.041).

Using the absolute classification of improvement as treatment response (Table 5), better outcomes were significantly associated with MOG-Ab positivity (OR = 0.26 versus AQP4-Ab positivity; 95% CI [0.12; 0.52]; *p* < 0.001). Poorer responses were significantly associated with delayed PE (OR = 1.04 per day; 95% CI [1.01; 1.06]; *p* = 0.00431) and extensive ON (OR = 4.82; 95% CI [1.80; 13.8]; *p* = 0.00315). Treatment response did not significantly correlate with bilateral ON (OR = 1.68; 95% CI [0.71; 3.97]; *p* = 0.24), nor ON associated with papillary edema (OR = 0.55; 95% CI [0.21; 1.39]; *p* = 0.22), nor longitudinally extensive transverse forms of myelitis (OR = 0.93; 95% CI [0.41; 2.2]; *p* = 0.86). At the multivariate analysis, MOG-Ab positivity was significantly and independently associated with better responses (OR = 0.048 vs AQP4-Ab positivity; 95% CI [0.0041; 0.35]; *p* = 0.008) and delayed PE with poorer responses (OR = 1.03 per day; 95% CI [1.01; 1.07]; *p* = 0.024).

## Discussion

We studied 211 NMOSD attacks in 183 long-term immunotherapy-naïve patients, their treatment, and their outcome. Most patients had a good response to the attack treatments at 6 months (63.5%), but a marked proportion did not respond (23.7%), which confirms the severity of NMOSD attacks and the need to optimize their treatment. Our analysis highlighted two associations of predictive interest, namely, the serogroup and the PE initiation delay.

This study investigated the first attacks of patients with an NMOSD presentation according to their serostatus. The strength of our study lies in the nationwide coverage of patient recruitment, the MRI confirmation of almost all attacks and the high time resolution of EDSS scoring, enabling a precise measure of recovery (relative definition) and treatment response (absolute definition). The significant result of our study was the prognostic implication of the serogroup. We showed that patients in the NMOSD–MOG+ group had a better prognosis than patients in the NMOSD–AQP4+ group. This is in line with the MOGADOR study, which showed that MOG+ patients had an overall better outcome, fewer relapses, and less frequently reached an EDSS score ≥ 3 than NMOSD–AQP4+ patients [8]. The differences between serogroups (age at onset, sex ratio, attack characteristics, evolution) likely result from pathophysiological specificities, both in terms of antigenic (MOG or AQP4) and cell type targeting (oligodendrocytes for NMOSD–MOG+ and astrocytes for NMOSD–AQP4+). The pathways involved in the pathogenesis associated with NMOSD–MOG+ damage CNS myelin in a complement-independent manner, without leading to immune cell infiltrates, and appear to yield more reversible neural dysfunctions [24, 25]. These observations could have practical implications for personalizing attack treatment regimens based on the serogroup. However, this information is not currently available in the days immediately after the attack onset.

Our results are nevertheless already applicable to a clinical situation corresponding to a first attack in a patient with an NMOSD presentation. It should be noted that in such cases a decision on the therapeutic strategy needs to be made even though information on the patient’s serostatus is not immediately available. Although the treatment regimen in our French population was relatively homogeneous, with corticosteroids as first-line therapy and PE as second-line therapy, our study highlighted that delayed PE initiation was detrimental. PE are currently considered as an adjunctive therapy for NMOSD attacks. We argue that PE should be used systematically and combined with corticosteroids as soon as possible right from the first attack if its characteristics are suggestive of NMOSD. Currently, some an expert opinion [14] and a set of guidelines [26] suggest the use of PE right away for subsequent attacks in patients who did not respond to corticosteroids in the past. However, apart from our study, several reports have shown that early PE are associated with better recoveries, regardless of disease modifying treatments. In 2018, Bonnan et al*.* found that the probability of achieving a complete recovery continuously decreased as the delay to PE increased: from 50% when started at day 0 to 1–5% when started after day 20 [20]. In their cohort of 115 severe NMOSD attacks (AQP4+ or seronegative), the median PE delay was 7.0 days, in most cases associated with corticosteroids, and 63% of patients received PE within 10 days after attack onset, making this population different from ours (median delay to PE in our study: 16 days). Likewise, investigating a subgroup of 207 NMOSD attacks treated by PE, Kleiter et al*.* found 40% of complete recovery when PE was started within 2 days after symptoms onset, 29% within 6 days, with a rapid decrease beyond as there were no cases of complete recovery when PE delay exceeded 20 days [18]. Despite longer delays to PE in our cohort the effect of the delay to PE remained significant. This suggests that PE should be considered for severe attacks even at a late stage after the onset of symptoms. No such associations with the other lines of treatments have been found. A retrospective comparison further encouraged the combination of PE with corticosteroids as an attack treatment rather than corticosteroids alone [27]. IA could not be studied in our population. IA is an alternative apheresis therapy, which selectively depletes immunoglobulins without requiring transfusion of another patient’s plasma [28]. This extracorporeal technique has been less studied than PE, but shows a similar efficacy in small heterogeneous populations with neuroinflammatory diseases [29, 30]. We did not find predictive associations for recovery with age or attack location, in contrast to the recent study of NMOSD attacks in the LATAM registry [21]. Some discrepancies with our study might be due to geographic population differences and patients not always being treatment naïve. The different serostatuses were analyzed separately, whereas we considered them as predictors. In our study, NMOSD–AQP4+ correlated with greater ages at onset (Table 1; *p* = 0.021) and with poorer outcomes, which suggests a confusion bias between age at onset and outcome. Finally, the delays to treatments were pooled, whereas we analyzed them individually. Considering the rapid distribution of intravenous methylprednisolone and its short half-life of 0.25 h [31], infusions could be planned at return of PE cycles sessions or every other day between PE cycles to avoid its clearance through apheresis. Thus, early initiation of PE in combination with corticosteroids seems to be a valuable therapeutic strategy for the first NMOSD attack but should be assessed more deeply in a clinical trial. The early adjunction of PE to treat subsequent attacks is more a matter of discussion given the better prognosis of NMOSD–MOG+ and the potential contributory effect of disease modifying treatments.

This retrospective cohort study has limitations inherent in its design, including the retrospective EDSS scoring, when the EDSS score was not available prospectively, indications bias in the choice of attack treatments and confusion bias. However, by selecting treatment-naïve patients, we avoided confusion with the anti-inflammatory effect of long-term immunotherapy treatments. By selecting the first attack or the next in an independent location, we avoided the risk of confusion with pre-existing disability. Contrary to recent randomized clinical trials, the attacks were physician-defined rather than adjudicated [32–34]. Strict attack definitions with severity thresholds and adjudications are better suited to prospective time-to-event studies. However, most of these definitions are based on the functional system scores or MRI data, which were the modalities used in our study. Physician-defined attacks are more representative of real-world practice and better suited to retrospective studies. In addition, as in phase 3 clinical trials, the diagnosis and the clinical course of each patient was reviewed by a panel of NMOSD experts belonging to the NOMADMUS scientific committee. The EDSS score has some limitations in assessing disability in NMOSD. It was designed to evaluate disability in patients with MS. It is non-linear and the dominance of the motor function may mask impairments of other systems when EDSS score ≥ 5.5. Unfortunately, there is no better assessment of disability available today for NMOSD and this approach is still used in phase 3 trials. The stratification into three outcome classes attenuated classification bias related to the retrospective EDSS scoring. The risk of potential misclassification was low according to the relative definition, likely to reduce the whole recovery class, and moderate according to the absolute definition, which was conversely sensitive to slight changes in EDSS score. This stratification aimed to evaluate more precisely the functional improvement in the “partial recovery” class (EDSS score at 6 months between the EDSS score at the nadir of the attack and a complete return to the previous state). The absolute definition is intended to create a more relevant evaluation tool to assess the treatment response. Finally, the early use of recently approved treatments, such as eculizumab, satralizumab and inebilizumab [32–34], could not be assessed as the disease onset in our population was their investigation and approval. The relationship between the recovery of the first attack and the long-term prognosis is another question of interest.

## Conclusions

This national collaborative study sheds light on the therapeutic management of NMOSD presentations. We identified two prognostic factors: serostatus (with better outcomes among MOG-Ab-positive patients) and the delay to PE. We, therefore, argue in favor of more aggressive anti-inflammatory management, requiring early combination of PE with corticosteroids. Furthermore, the lower severity and better treatment response of NMOSD–MOG+ support its nosologic reclassification as MOGAD.

## Data Availability

Anonymized data will be shared by request from any qualified investigator.

## Abbreviations

Ab: Antibody
ADEM: Acute disseminated encephalomyelitis
AQP4: Aquaporin-4
CNIL: Commission Nationale de l'Informatique et des Libertés
CNS: Central nervous system
EDSS: Expanded Disability Status Scale
IPND: International Panel for NMO Diagnosis
IVIG: Intravenous immunoglobulins
MOG: Myelin oligodendrocytes glycoprotein
MOGAD: MOG associated disease
MRI: Magnetic resonance imagery
MS: Multiple sclerosis
NMO: Neuromyelitis optica
NMOSD: Neuromyelitis optica spectrum disorders
NMOSD–AQP4+: Neuromyelitis optica spectrum disorders with anti-AQP4 antibodies
NMOSD–DN: Neuromyelitis optica spectrum disorders with double negative serologies
NMOSD–MOG+: Neuromyelitis optica spectrum disorders with anti-MOG antibodies
OFSEP: Observatoire Français de la Sclérose En Plaques
OR: Odds ratio
PE: Plasma exchanges
SD: Standard deviation
